# Hiring, training, and supporting Peer Research Associates: Operationalizing community-based research principles within epidemiological studies *by*, *with*, and *for* women living with HIV

**DOI:** 10.1186/s12954-019-0309-3

**Published:** 2019-07-18

**Authors:** Angela Kaida, Allison Carter, Valerie Nicholson, Jo Lemay, Nadia O’Brien, Saara Greene, Wangari Tharao, Karène Proulx-Boucher, Rebecca Gormley, Anita Benoit, Mélina Bernier, Jamie Thomas-Pavanel, Johanna Lewis, Alexandra de Pokomandy, Mona Loutfy, On behalf of the CHIWOS Research Team, Rahma Abdul-Noor, Aranka Anema, Jonathan Angel, Dada Mamvula Bakombo, Fatimatou Barry, Greta Bauer, Kerrigan Beaver, Marc Boucher, Isabelle Boucoiran, Jason Brophy, Lori Brotto, Ann Burchell, Claudette Cardinal, Allison Carter, Lynne Cioppa, Tracey Conway, José Côté, Jasmine Cotnam, Cori d’Ambrumenil, Janice Dayle, Erin Ding, Danièle Dubuc, Janice Duddy, Mylène Fernet, Annette Fraleigh, Peggy Frank, Brenda Gagnier, Marilou Gagnon, Jacqueline Gahagan, Claudine Gasingirwa, Nada Gataric, Rebecca Gormley, Saara Greene, Danielle Groleau, Charlotte Guerlotté, Trevor Hart, Catherine Hankins, Emily Heer, Robert S. Hogg, Terry Howard, Shazia Islam, Joseph Jean-Gilles, Hermione Jefferis, Evin Jones, Charu Kaushic, Mina Kazemi, Mary Kestler, Maxime Kiboyogo, Marina Klein, Nadine Kronfli, Gladys Kwaramba, Gary Lacasse, Ashley Lacombe-Duncan, Melanie Lee, Rebecca Lee, Jenny Li, Viviane Lima, Elisa Lloyd-Smith, Carmen Logie, Evelyn Maan, Valérie Martel-Lafrenière, Carrie Martin, Renee Masching, Lyne Massie, Melissa Medjuck, Brigitte Ménard, Cari L. Miller, Judy Mitchell, Gerardo Mondragon, Deborah Money, Ken Monteith, Marvelous Muchenje, Florida Mukandamutsa, Mary Ndung’u, Valerie Nicholson, Kelly O’Brien, Nadia O’Brien, Gina Ogilvie, Susanna Ogunnaike-Cooke, Joanne Otis, Rebeccah Parry, Sophie Patterson, Angela Paul, Doris Peltier, Neora Pick, Alie Pierre, Jeff Powis, Karène Proulx-Boucher, Corinna Quan, Jesleen Rana, Eric Roth, Danielle Rouleau, Geneviève Rouleau, Sergio Rueda, Kate Salters, Margarite Sanchez, Roger Sandre, Jacquie Sas, Édénia Savoie, Paul Sereda, Stephanie Smith, Marcie Summers, Wangari Tharao, Christina Tom, Cécile Tremblay, Jason Trigg, Sylvie Trottier, Angela Underhill, Anne Wagner, Sharon Walmsley, Clara Wang, Kath Webster, Wendy Wobeser, Denise Wozniak, Mark Yudin, Wendy Zhang, Julia Zhu

**Affiliations:** 1 Faculty of Health Sciences, Simon Fraser University, Blusson Hall Room 10522, 8888 University Drive, Burnaby, BC V5A 1S6 Canada; 2 British Columbia Centre for Excellence in HIV/AIDS, Vancouver, BC Canada; 3 Faculty of Medicine, Kirby Institute, University of New South Wales, Sydney, NSW Australia; 4 Canadian Aboriginal AIDS Network, Vancouver, BC Canada; 5 Positive Living British Columbia, Vancouver, BC Canada; 6 Chronic Viral Illness Service, McGill University Health Centre, Montreal, QC Canada; 7 Department of Family Medicine, McGill University, Montreal, QC Canada; 8 School of Social Work, McMaster University, Hamilton, ON Canada; 9 Women’s Health in Women’s Hands, Toronto, ON Canada; 10 Women’s College Research Institute, Women’s College Hospital, Toronto, ON Canada; 11 Dalla Lana School of Public Health, University of Toronto, Toronto, ON Canada; 12 Faculty of Nursing, University of Montreal, Montreal, QC Canada; 13 Department of History, York University, Toronto, ON Canada; 14 Faculty of Medicine, University of Toronto, Toronto, Canada

**Keywords:** HIV, Women, Community-based research, Training, Peers, Harm reduction, Community engagement, Cohort studies, Canada, CHIWOS

## Abstract

**Background:**

A community-based research (CBR) approach is critical to redressing the exclusion of women—particularly, traditionally marginalized women including those who use substances—from HIV research participation and benefit. However, few studies have articulated their process of involving and engaging peers, particularly within large-scale cohort studies of women living with HIV where gender, cultural and linguistic diversity, HIV stigma, substance use experience, and power inequities must be navigated.

**Methods:**

Through our work on the Canadian HIV Women’s Sexual and Reproductive Health Cohort Study (CHIWOS), Canada’s largest community-collaborative longitudinal cohort of women living with HIV (*n* = 1422), we developed a comprehensive, regionally tailored approach for hiring, training, and supporting women living with HIV as Peer Research Associates (PRAs). To reflect the diversity of women with HIV in Canada, we initially hired 37 PRAs from British Columbia, Ontario, and Quebec, prioritizing women historically under-represented in research, including women who use or have used illicit drugs, and women living with HIV of other social identities including Indigenous, racialized, LGBTQ2S, and sex work communities, noting important points of intersection between these groups.

**Results:**

Building on PRAs’ lived experience, research capacity was supported through a comprehensive, multi-phase, and evidence-based experiential training curriculum, with mentorship and support opportunities provided at various stages of the study. Challenges included the following: being responsive to PRAs’ diversity; ensuring PRAs’ health, well-being, safety, and confidentiality; supporting PRAs to navigate shifting roles in their community; and ensuring sufficient time and resources for the translation of materials between English and French. Opportunities included the following: mutual capacity building of PRAs and researchers; community-informed approaches to study the processes and challenges; enhanced recruitment of harder-to-reach populations; and stronger community partnerships facilitating advocacy and action on findings.

**Conclusions:**

Community-collaborative studies are key to increasing the relevance and impact potential of research. For women living with HIV to participate in and benefit from HIV research, studies must foster inclusive, flexible, safe, and reciprocal approaches to PRA engagement, employment, and training tailored to regional contexts and women’s lives. Recommendations for best practice are offered.

## Plain English Summary

Engaging affected communities in health research is increasingly recognized as key to improving the relevance of research and care. However, moving principles into practice is challenging, particularly in harm reduction and HIV fields where stark social inequalities can impact the process of engagement. In this paper, we describe a national approach of hiring, training, and supporting women living with HIV from drug using and non-drug using communities as Peer Research Associates (PRAs) in a large cohort study that has enrolled and surveyed 1422 women living with HIV in three Canadian provinces (British Columbia, Ontario, and Quebec). Our process included (1) creating a hiring team comprised of members representing research, healthcare, and women living with HIV; (2) implementing employment equity and diversity practices to ensure representation of women with diverse research backgrounds and identities and opportunities for community capacity building; and (3) designing and implementing a comprehensive experiential research training curriculum that gave equal weight to lived experience in relation to other knowledge bases and prioritized “learning by doing.” Over 40 women living with HIV were engaged as PRAs over a 7-year period. Challenges in hiring, training, and supporting PRAs included navigating shifting roles from community member to researcher; effectively responding to women’s multiple and varied social locations; providing on-going support through personal and professional struggles; and study time and resource constraints. The benefits of this approach included building capacity for both PRAs and researchers, recruiting traditionally “harder-to-reach” study participants, and building strong community partnerships with local and national organizations.

## Background

Women now represent over half of the estimated 37 million people living with HIV worldwide [1]. In Canada, women comprise approximately one quarter of all people living with HIV, accounting for 16,600 women [2]. HIV prevalence, incidence, and impact among women are inequitably distributed by several social factors, including poverty, injection drug use, and/or sex work history, incarceration history, refugee and newcomer status, ethnicity (e.g., Indigenous; African, Caribbean, or Black), and lesbian, gay, bi, trans, queer, or two-spirit (LGBTQ2S) identity, with several points of intersection between and within these groups [2–4].

In particular, substantial overlap exists between communities of women living with HIV and women who use illicit drugs. Nearly one quarter (22%) of women living with HIV in Canada acquire HIV through injection drug use [5], and an estimated 10% of women who currently inject drugs are living with HIV [6]. Moreover, the prevalence of current illicit drug use among women living with HIV (i.e., 16.8% report regular crack/cocaine use and 11.3% report regular/occasional heroin use) is several magnitudes higher than the estimated 0.1% prevalence reported among the general population of Canadian women of similar age and ethno-racial profiles [7].

While modern antiretroviral therapy (ART) has dramatically lowered mortality, morbidity, and transmission risk for people living with HIV as a whole [2, 8–10], significant gender-based differences in health outcomes persist [11–14]. Compared with men, women are diagnosed at more advanced disease states, have longer delays in initiating ART [15–18], are more likely to receive poorer quality of care [19], and are less likely to achieve virologic suppression [20]. Poorer outcomes across the HIV care cascade extend to inequities in life expectancy. At age 20, women livign with HIV in Canada have a remaining life expectancy of 32.4 years, which is 6.8 years shorter than the remaining life expectancy of men living with HIV (39.2 years). [21]. Inadequate access to care is even more pronounced for women with manifold historical and social disadvantages, including Indigenous and racialized women, transgender women, and women who inject drugs [21, 22].

Despite awareness of women’s poorer HIV care and clinical outcomes, women living with HIV continue to be under-represented in HIV research. Within existing HIV studies that do involve women, there remains an over-emphasis on individual risk factor epidemiology, which is limited in its scope, understanding, and application to women’s health priorities. Such approaches seldom employ a gendered, racialized, or social lens to explore HIV research gaps, creating conditions whereby the most vulnerable communities of women affected by HIV are excluded from research participation and marginalized from research benefit. In response, there have been calls for a gendering of the “nothing about us without us” movement, including advocacy for increased opportunities for women to contribute to research, policy, and programming that impact their lives [23]. Within the HIV community, advocacy for inclusive approaches is articulated through the principles of the Greater Involvement of People living with HIV (GIPA) and its companion principle, the Meaningful Involvement of People living with HIV/AIDS (MIPA) [24]. At the 1994 Paris AIDS Summit, 42 governments including Canada endorsed the GIPA/MIPA principles [24]. However, after experiencing continued gender inequities in HIV clinical outcomes and the undervaluing of women in GIPA/MIPA movements, women living with HIV advanced a new principle: the Meaningful Involvement of Women living with HIV/AIDS (MIWA) [25].

These important movements have contributed to national and global statements that call for health science research to embrace community-based research (CBR) principles and engage affected community members in the research process. Such approaches are necessary to redress gendered and social marginalization from meaningful HIV research participation and benefit [23, 26–28]. CBR principles stipulate that intentional steps are taken to disrupt the power imbalances typically present between researchers and the community that is being researched by fostering collaborative, co-learning partnerships between community members and researchers [29]. In the context of research involving women living with HIV, expectations extend to an explicit commitment to the GIPA and MIWA principles, which require both involvement of women living with HIV throughout the research process and *meaningful engagement* as defined by those involved in the project [28, 30]. Such an approach promises to improve the understanding of the social context of disease and to contribute more meaningful and nuanced findings to the development of appropriate and accessible health programs and policies [26]. CBR offers a particularly promising opportunity for achieving these objectives because of the strong connection between multiple oppressions and health outcomes.

However, few epidemiological studies have articulated the process of meaningful community engagement in HIV research, particularly, within large-scale, national cohorts of women living with HIV where gender, cultural and linguistic diversity, HIV-related stigma, substance use stigma, and power inequities must be navigated. In this paper, we describe a national approach to CBR that involves hiring, training, and supporting women living with HIV to work as Peer Research Associates (PRAs) within the Canadian HIV Women’s Sexual and Reproductive Health Cohort Study (CHIWOS). We discuss the main challenges and opportunities encountered in this approach and offer recommendations for best practice towards informing future cohort studies involving women living with HIV, women who use drugs, and other underserved populations.

## Methods

### The CHIWOS project

CHIWOS is a multi-site, longitudinal, community-based research project conducted *by*, *with*, and *for* women living with HIV, in collaboration with allied researchers, service providers, policy-makers, and other stakeholders [31]. Between August 2013 and May 2015, we enrolled 1422 women living with HIV (trans- and cis-gender inclusive) across the three Canadian provinces where a majority of women living with HIV reside (British Columbia, Ontario, and Quebec) [2], using non-random, purposive sampling to recruit typically underserved women. The principal study aims were to assess the barriers and facilitators to use of women-centered HIV care and the impact of such patterns of use on reproductive, sexual, mental, and women’s health outcomes [32]. Participants completed a Peer Research Associate (PRA)-administered questionnaire (completion time of 90–120 min) at baseline, with 18- and 36-month follow-up visits [33]. The study is grounded in CBR principles [34] and guided by social determinants of health [35] and critical feminism [36] frameworks, integrating principles of anti-oppression, social justice, and intersectionality [37]. Overall, we aimed to produce meaningful research that is community-driven and used to enact change to support the health of women living with HIV in Canada. CHIWOS methods are described in detail elsewhere [31, 33].

### The CHIWOS approach to peer engagement in research

CHIWOS operationalized MIWA through hiring, training, and supporting women living with HIV to engage in the study as PRAs [25, 27]. In CHIWOS, PRAs are self-identified women living with HIV (cis- and trans-inclusive) who share social identities (e.g., Indigenous, racialized, sexual minority, and trans women) and lived experiences (e.g., injection drug use, sex work, incarceration, childhood and adulthood violence experiences) with the community of women living with HIV in Canada, and who are familiar with the local HIV-related health and social care priorities for women. PRAs were engaged as equal partners in all stages of the research process: from defining the research question and priorities [33], designing and piloting the survey questions [38], through to participant recruitment [39], data collection and analysis, and the dissemination of findings (including delivering presentations and workshops, co-authoring manuscripts, and engaging with traditional and online media). What follows is a discussion of our approach to meaningfully engaging PRAs in CHIWOS and the challenges and opportunities that emerged.

## Results

### Operationalizing peer involvement principles and practices

#### Hiring Peer Research Associates

To ensure that diverse communities of women living with HIV were meaningfully included in the research, the PRA hiring process adhered to the following core principles: (1) prioritize the engagement of women historically under-represented in research, including women from racialized, Indigenous, LGBTQ2S, drug use, sex work, and rural communities; (2) value lived experience as an important form of knowledge to inform and strengthen the research; and (3) acknowledge that PRAs may not have previous research or formal employment experience and remain committed to capacity building when hiring women with a range of research, employment, and lived experience.

To operationalize these principles, an *interview and hiring panel* was formed in each province with membership from stakeholders representing a mix of skills, roles, and experience to ensure that the hiring process was supportive, inclusive, and accessible. Panels included the provincial principal investigator, research coordinator, regional clinic and/or AIDS Service Organization (ASO) partner, and a woman living with HIV with previous CBR experience. Together, the members of the panel devised a recruitment strategy which aimed to foster team diversity and representation, a low-barrier application process, and appropriate interview questions that flagged community concerns or sensitivities and highlighted the ways in which lived experience could positively contribute to the research.

We *recruited* applicants by advertising through clinics, ASOs, community and peer networks, online (e.g., websites, Facebook, and Twitter), and other informal channels across each province. Recruitment of applicants adhered to the basic principles of CBR to support the involvement and capacity building of community members. The *job application process* included a plain-language and transparent job description that provided an overview of the CHIWOS project, a summary of the position duties and responsibilities, and specified the compensation and reporting details. Applicants were invited to submit a cover letter and resumé or to ensure the application process was not exclusionary for women who may not have had previous experience with formal hiring procedures, a brief, structured job application form with questions such as “Tell us about yourself” and “Please describe your interest and/or experience in HIV research.” In order to select a diverse team of PRAs, applicants were invited to describe the communities with which they identified. Where relevant, the job ad clearly specified any hiring strategies that reflected the region’s priorities. For instance, in British Columbia, a minimum of two PRA positions were prioritized for Indigenous women in Vancouver and Prince George, the two epicenters of HIV among Indigenous women in the province.

Nearly 70 applications were received across the three provincial sites. The members of the provincial interview and hiring panels independently reviewed all applications. Where appropriate, a shortlist of applicants was created (based on the hiring principles outlined above) and invited for in-person, video, or telephone interviews. *Interviews* were conducted using a scenario-based interview approach to many of the interview questions, which also allowed for the assessment of future training needs. The interview process created an early opportunity to consider ethical tensions and logistic challenges common to CBR studies, including PRA compensation strategies, comfort and safety of HIV disclosure, emotional risks and supports, and regional diversities.

Concluding the hiring and interviewing process, a national team of 37 PRAs was hired (8 in British Columbia, 20 in Ontario, and 9 in Quebec^1^) and included self-identified women from across geographic regions and with extensive diversity in terms of ethnicity, languages spoken, country of origin, age, gender identity, sexual orientation, and experiences of injection drug use and other substances, sex work, and incarceration. Consistent with our commitment to community capacity building, the PRA team presented with a range of lived and professional experiences, including women with experience working on other HIV CBR studies alongside women for whom this would be their first formal employment in research, as well as women with both advanced computer skills and no previous computer experience.

#### Rethinking peer researcher identities

Following the initial hiring process, provincial teams reflected on the notion of the peer researcher identity. Greene [40] previously highlighted the challenges associated with defining “peer,” arguing that people living with HIV are not a homogenous group, rather they carry different histories, identities, and social locations [41]. While we were seeking to hire peer researchers based on their identity as women living with HIV, we reflected that HIV serostatus may not be the most dominant or defining social identity. Rather, for many women, their identities in relation to current or former drug use, sex work, ethno-racial ancestry, sexual orientation, gender, or geographic community, were commonly a more defining peer identity than HIV serostatus. We responded to the complexity of the definition of a “peer” by opening the hiring process to recruit additional PRAs with identities and regions not previously well-represented in the initial PRA team. For instance, in Quebec, we received no applications from Indigenous or trans women living with HIV. Consistent with our reflection that HIV status may not be the most dominant social identity upon which participants might relate to PRAs and feel safe during the interview, we hired HIV-negative women working in ASOs who identified as Indigenous and trans, and who practiced allyship to women living with HIV. Through team discussions, we considered allyship to be an active practice, rather than a static identity, consistent with the definitions forwarded by other scholars on allyship [42].

#### Training Peer Research Associates

In response to the diversity of the national PRA team, the research team designed a tailored PRA training curriculum alongside a system of on-going support and continuing mentorship opportunities, as a strategy to operationalize a commitment to community capacity building. A national PRA Training Committee comprised of various members of the research team including researchers, CBR experts, and women living with HIV, many of whom had experience developing and implementing PRA training in other CBR studies [41, 43], was formed to design the PRA training curriculum. The committee acknowledged that developing a rigorous adult training curriculum demands expertise, and hired a curriculum developer with extensive experience in community-building and education projects in HIV (JL) to help guide the process. Together, the PRA Training Committee and JL engaged in a collaborative process over several months to design, develop, and implement a national, bilingual, comprehensive, multi-phase, evidence-based training curriculum grounded in experiential and adult learning principles [44].

We began by brainstorming the critical topics for inclusion in training. This was an inductive process, which began with outlining the entry skills and attributes of our team of hired PRAs and the intended training outcomes, then working backwards to identify and prioritize the core training concepts, knowledge, skills, assessment approaches, and appropriate teaching methods. This was an important step in our approach to ensure that the training complemented PRAs’ lived experience and was tailored to their specific needs. We also reviewed existing excellent online PRA research training resources (e.g., the Ontario HIV Trial Network’s Learning Exchange for Peer Researchers in HIV/AIDS (LEAP), now housed in Universities Without Walls) [45] and prior research on building health research capacity of PRAs [41, 46] to inform our training curriculum. An overview of the PRA Training Outcomes guide is included in Table 1.

**Table 1:**
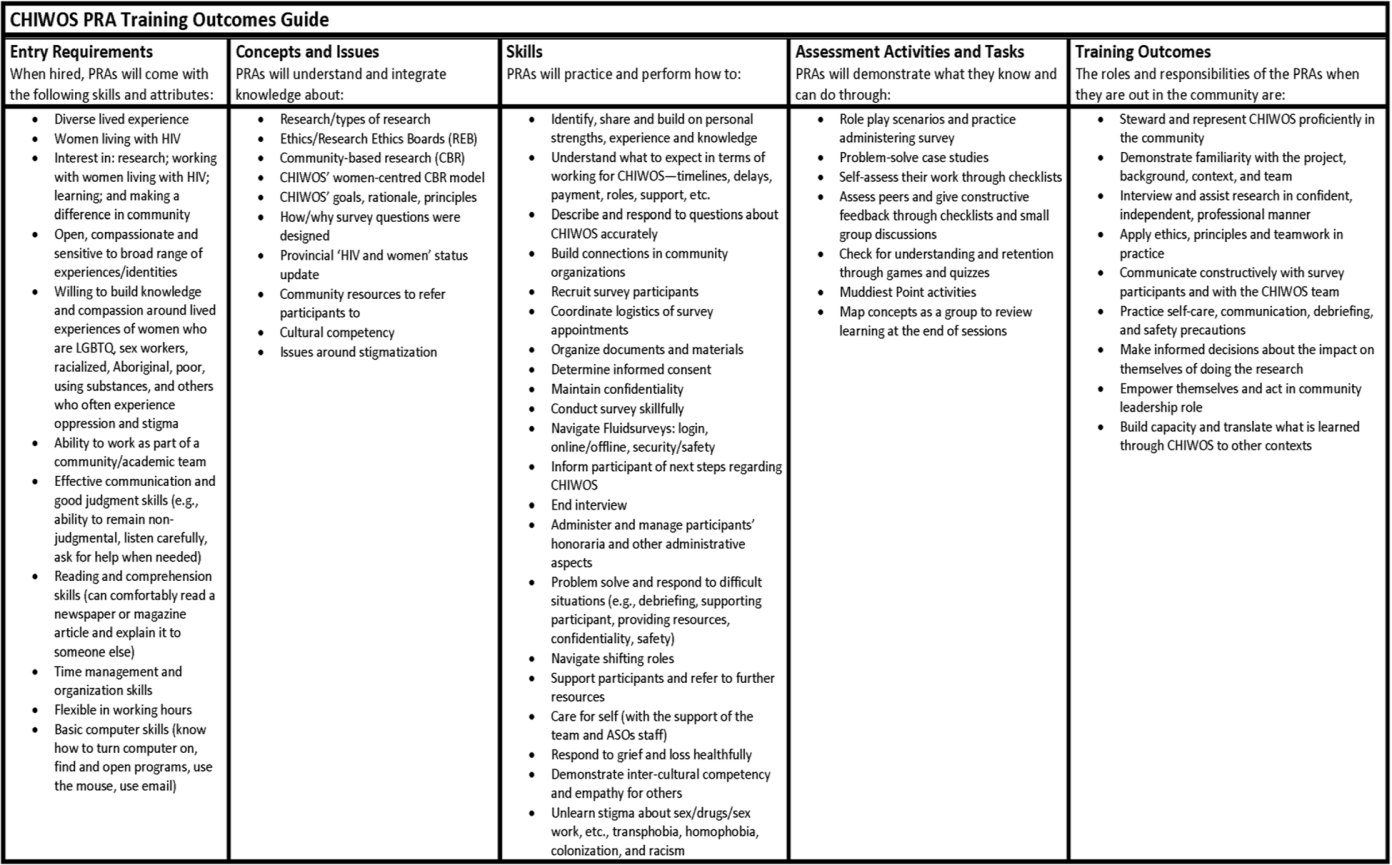
Outcomes guide for the CHIWOS study Peer Research Associate (PRA) training curriculum

Training was delivered across two 2-day workshops (in British Columbia and Quebec) and one 3.5 day workshop in Ontario. As PRAs in Quebec are unilingual French or English, these workshops were conducted bilingually, while in Ontario, bilingual facilitators paused for clarification when needed. As several PRAs had young children, the training was child-inclusive. For example, in British Columbia, the PRA training committee made arrangements to enable one PRA to bring her young child to the training sessions and to support her to complete all training sessions accordingly.

PRA compensation practices must be made within the financial, ethical, and legal considerations of the institutions and funding agencies involved. However, given a tendency for peer work to be undervalued and poorly enumerated, it is important to be transparent about compensation policies employed in community-based research. For our training, PRAs were financially compensated for the time spent engaged in the training curriculum. All PRA food, accommodation, and travel expenses (including costs associated with childcare for those PRAs whose children did not accompany them to the training) were covered. Each PRA was paid an honorarium of $100 per day spent in training (i.e., for 4 days of training they received a $400 honorarium). PRAs were paid the same amount, regardless of previous research experiences or circumstances. PRAs were provided $50 per day for childcare as needed. Decisions regarding payment amounts and procedures were made in consultation among team members, including PRAs with previous CBR experience, academic researchers, and ASO staff members, and were informed by published recommendations on PRA compensation [47].

All training materials (i.e., presentations, PRA binders, handouts) were made available in both French and English. In addition, PRAs were invited to attend other provincial trainings; for instance, a Francophone PRA from Ontario attended both the Quebec and Ontario training to benefit from both the teachings and team building aspects of the trainings. Ensuring all materials were available in both official languages, skilled bilingual training facilitators and structured small group activities allowing for French and English subgroups were crucial to the implementation of a language-inclusive training curriculum.

An overview of the 4-day training agenda is provided in Table 2. The complete PRA training guide and facilitator’s handbook are available on the CHIWOS website as open access documents, in both in English and French [49]. The PRA training curriculum was implemented provincially and tailored to regional contexts. Traditional topics related to research methods were included in the curriculum, including CBR principles, administering quantitative surveys, skillful interviewing practices, and ethical standards, carefully emphasizing and outlining the importance of confidentiality and informed consent. Support with computer literacy was also provided to help PRAs effectively use study-provided laptops and navigate our online survey data capture tools.

**Table 2:** Overview of the CHIWOS Peer Research Associate training agenda, curriculum, and teaching activities

	Day 1	Day 2	Day 3	Day 4
Morning	Welcome and opening •Acknowledgement of Indigenous territories •Introductions via team strengths scavenger hunt and map •Learning outcomes and agenda	Welcome and opening: •Acknowledgement of Indigenous territories •Overview of the agenda •Clarifying ^“muddiest points”^	Welcome and opening: •Acknowledgement of Indigenous territories •Overview of the agenda •Clarifying “muddiest points” •Project details scavenger hunt	Welcome and opening: •Acknowledgement of Indigenous territories •Overview of the agenda •Clarifying “muddiest points” •Developing a study “elevator speech”
	Orientation to study: •Study background •Provincial epidemiology of HIV and women •Study justification and goals •Study guiding frameworks and principles •Study design and timeline Orientation to the job of a Peer Research Associate: •What to expect in working with CHIWOS	Successful surveying: •Brainstorm skillful/unskillful survey practices •Survey demonstration and discussion •Rationale of survey design	How to’s: •Overview of binder contents and HR agreements •Overview of the job of a PRA •Participant recruitment •Appointment logistics •What to bring to conduct a survey	Survey rationale: •Purpose and rationale behind each survey question Informed consent process: •Demonstration and practice Closing a survey: •Resources for participants •Demonstration and practice
Lunch and team building activity				
Afternoon	Roadmaps for connection: •Poem and discussion: *Turning to One Another* [48] •Storytelling roadmap activity to create and discuss roadmaps for “bridging our differences,” “unlearning prejudices,” “self-care,” “changing roles”	Hands-on survey practice and feedback	Safety and well-being: •Supports for participants and PRAs •Emotional and psychological precautions •Boundaries and triggers •Self-care plans Problem-solving scenarios: •Role playing	Introduction to the online survey data capture interface: •Training and hands-on practice
	Closing: •Review: concept map •Plan for next day •Evaluation: muddiest point •Closing round: “one idea I’m leaving with”	Closing: •Concept mapping: debrief on survey process and Experiences •Plan for the next training •Closing round: impact of the day on the “head, heart, and hands”	Closing: •Review: concept map •Plan for the next day •Evaluation: muddiest point •Closing round: “one idea I’m leaving with”	Closing: •Next steps for training and starting the job (e.g., survey piloting, team meeting) •Closing round: impact of the training on the “head, heart, and hands”

Note: Teaching activities were delivered by a range of team members including Peer Research Associates, the study principal investigators, project coordinators, and an expert in adult education

In light of a number of methodological and ethical tensions raised by other HIV CBR studies that include PRAs [40, 41, 50], training on social positioning, traversing multiple roles in the community (i.e., as peer, client, patient, researcher), the diversity of study participants, unlearning prejudices, self-care and support for participants and PRAs, and challenging interview scenarios were incorporated in the original training process, as well as in ongoing annual training workshops. Finally, opportunities to support team building, trust, and safety were incorporated into the training curriculum, including icebreakers, informal team dinners, training on self-care and PRA-driven guidelines for creating safety in our learning environment and on our team (e.g., sharing space, contributing stories that enhance work and learning, practicing self-care).

Building on experiential learning theory [44], the research team held that the most appropriate way to prepare PRAs for the realities of conducting research was to “learn by doing” and by reflecting on their experiences, actions, and outcomes: not only as a PRA, but also as a research participant. As such, training in the above curricula was facilitated with a number of experiential learning activities and critical reflection periods. The training involved a combination of teaching methods, including short presentations, large and small group discussions, live demonstrations, role playing with opportunities for feedback, story road mapping, elevator speeches to gain comfort in describing the study and their role, and “muddiest point” exercises to monitor learning. A collaborative, strength-based approach to training was also employed. All team members, including PRAs, co-facilitated various training activities, which brought both professional and lived experience [44] to the process and added depth, poignancy, and relevancy to the topics covered.

#### Ongoing supervision, mentorship, and support of Peer Research Associates

As PRA training is an iterative, non-linear process, on-going supervision, mentorship, and support were woven throughout the research process using several strategies. Before going into the field, each PRA conducted interviews with other PRAs as an opportunity to safely practice the entire interview process. Each PRA was paid $125 to complete two practice interviews, one serving as the interviewer (at a rate of $75) and the other serving as the participant ($50). Once in the field, for many PRAs, their first few interviews were conducted with women in their close networks in order to build confidence and address any survey glitches. As enrolment progressed, PRA team meetings were held monthly, or as needed, to foster continual learning, support, and team-building. As part of these meetings, data quality and recruitment reports were presented and discussed with PRAs to troubleshoot study challenges such as varying interpretations of survey questions and difficulties with recruiting certain populations. Regular one-on-one check-ins with a PRA and the research coordinator and/or principal investigator to debrief, discuss concerns, ensure data quality, and address individual training needs were held as necessary. Other strategies to ensure PRA (and participant) support included the following: hiring an on-call study counsellor (who was accessible free-of-charge to the PRA and anonymously, if preferred), creating self-care resource brochures, and developing partnership agreements with local clinics and ASOs across each province for in-person support, which was especially critical for remotely located PRAs. Lastly, we developed a private, PRA learning hub with interactive training modules, a discussion forum for learning, and a space to connect with PRA colleagues from across Canada. Anticipating PRA turnover, this training website also allowed for offsite, flexible, and individualized training across the entire curriculum for new PRAs as well as non-PRA allies within the research team. Yearly “refresher” training workshops were held to revisit pertinent issues, to train and pilot for the follow-up visit surveys, and to continue building PRA skills and capacity that extend beyond survey administration.

### Challenges encountered and responses to operationalizing peer involvement principles and practices

Previous research has identified key challenges to PRA training within the context of a CBR study, including limited financial resources and a significant time commitment required to sustainably support trainees [43, 51, 52]. We were fortunate to learn from this work to develop and implement a PRA hiring and training process. Although our process was successful in identifying and building capacity of our team to lead participant recruitment and data collection responsibilities of this CBR study, we encountered a number of additional challenges that demanded our attention at different points in the process. In this section, we outline these challenges, highlight strategies employed to address these challenges, and acknowledge that overcoming these challenges is an ongoing process.

#### Remaining responsive to the range of PRA skills and experiences

For some CHIWOS PRAs, this was the first time that they were involved in research and/or the first time using online survey software and computers for professional purposes, while others had worked as research associates on previous studies. This meant that some of our PRAs experienced a steep learning curve, which has been noted as a potentially frustrating process in other CBR projects [41]. To overcome these challenges in CHIWOS, we started training with research basics and found that a team- and strength-based approach enabled PRAs to help support each other in their differences in skills. We incorporated team-bonding activities including team dinners, icebreakers, and group reflections as different means of developing trust as a group. Greene et al. [41] echoed this approach and similarly found that developing group cohesion through trust and strength had a positive impact on the PRA training process. Developing training with the needs of PRAs from diverse backgrounds in mind and developing opportunities for one-on-one learning were also ways in which we accounted for the range of women’s skills and experiences.

#### Ensuring PRA safety, health, and well-being

Emotional well-being had to be prioritized given the potential for training materials and topics to be triggering of personal experiences. Thus, we developed a team protocol for debriefing with PRAs throughout training and while in the field, as well as ensuring that confidential counseling by a certified professional was freely available and accessible to PRAs. Lazarus et al. [50] similarly recognized the need to remain mindful of the emotional impact CBR can have on PRAs and took this into account in their training through support and ongoing debriefings. Furthermore, training can be physically taxing on PRAs, who are expected to participate in multi-day workshops with long training days. Providing activity and nutrition breaks and check-ins throughout training days was important to support PRA health and well-being. At times, this meant that certain activities had to be placed on hold or re-imagined to allow for adequate check-ins and breaks.

#### Ensuring PRA confidentiality and privacy relating to HIV status and other personal factors

For some women, the job title of a “PRA” itself presents a risk of involuntary and/or forced HIV disclosure. In CHIWOS, PRAs were not required to disclose their HIV status to participants, and some chose to work under pseudonyms to help protect their identities. Furthermore, consistent with other studies [50], PRAs had the option of declining to interview participants that they knew outside the study and/or to interview participants over the phone or Skype rather than in person to avoid public disclosure as a person living with HIV. Limiting other threats to PRA confidentiality and privacy proved more challenging. For instance, in focus groups with PRAs working in HIV CBR research, Cain et al. [53] reported that being identified as a “peer” introduced a burden and pressure to disclose HIV status or other personal characteristics to participants when PRAs did not feel safe to do so. Moreover, if a PRA does decide to share personal information with participants, there is a little control over what the participants may then share with others in the community [41].

#### Managing tensions around PRAs’ shifting roles from the community member to PRA and study steward

As many CHIWOS PRAs are active in their respective communities, tensions arose when women shifted from a “friend” of a participant to a “PRA,” navigating different roles in their relationships. Additionally, and consistent with previous findings [41], it can be challenging for PRAs who want to intervene and help a participant who may be struggling, to remember their role as a PRA, and to maintain professional and personal boundaries. It can also be challenging for PRAs to acknowledge and address personal biases in their shifting roles. For instance, both PRAs with and without experience with drug-using communities expressed forms of bias about recruiting women engaged in substance use within the study, including, for example, concerns about whether and how to navigate the survey procedures with an intoxicated participant. To support PRAs, the team collectively developed a detailed document outlining “guidelines for problem-solving challenging interview scenarios” [54]. This document reviewed common and possible scenarios that interviewers may face conducting the interviews with study participants and outlined the CHIWOS policies for how to address these scenarios. Throughout the guidelines, we emphasized that PRA safety and well-being were of paramount importance, moreso than completing an interview. Providing ongoing training and additional workshops led by professionals with lived and learned experience in setting and maintaining boundaries, unlearning prejudices, and practicing self-care were important training additions to continue supporting CHIWOS PRAs in the field.

#### Dedicating sufficient time and resources to translation

As a national study committed to a bilingual policy, we often underestimated timelines as well as budgets for translations and adaptations of materials. Implementing a fully bilingual PRA training (undertaken in Quebec) necessitated skilled bilingual facilitators and additional time and resources to ensure the quality of the training and team building opportunities.

#### Managing relationships between training facilitators and PRAs

Many PRAs have complex life situations, and facilitators needed to determine their appropriate role as a source of support in a professional manner. We navigated this by maintaining a flexible training approach to each PRA’s unique situation (e.g., welcoming PRAs to bring their young children to the training) and by ensuring that appropriate outreach (such as an anonymous on-call counselor) was available to all team members, including training facilitators.

#### Maintaining positive relationships with PRA applicants who were not hired

In British Columbia and Ontario, the hiring committees received more applications than available PRA positions. Provincial coordinators connected personally with each applicant who was not selected for the position to identify other opportunities to remain involved in CHIWOS, and to encourage on-going linkages with the core study team. Such options included participating in the CHIWOS Community Advisory Board, following CHIWOS on social media, and attending community events.

## Discussion

### Opportunities and lessons learned

By engaging in a collaborative and community-based approach to hiring and training, we were able to recruit a diverse group of 37 PRAs across Canada and support their involvement in meaningful engagement across various stages of the study. This model contributes to the growing literature on the involvement and meaningful engagement of peers in research. In addition, despite the highlighted challenges, hiring, training, and supporting a national team of PRAs offered numerous advantages and opportunities for CHIWOS that we believe will also be beneficial for other community-based researchers committed to the meaningful engagement of people with lived experience on research teams. These opportunities include:

*Overall team capacity building*. A diverse collaborative team generates genuine opportunity for different ways of knowing and mutual learning and growth of the whole team. The collaborative training process generated the co-production of new knowledge and growth for PRAs and researchers alike and allowed PRAs to define what meaningful engagement meant to them. For some participants, their PRA role included participation in manuscript development and presentations as well as the administration of surveys. PRAs who had additional employment were able to complete CHIWOS surveys as they fit within their existing schedule. In addition, the development of computer skills as well as access to laptops made it possible for PRAs to stay in touch with a geographically diverse team via email, social media, and Skype. Computer literacy skills also opened up additional employment opportunities for PRAs within and outside of research.

*Co-production of innovative solutions to study challenges*. The commitment to capacity and team building yielded secondary study benefits, including the co-creation of solutions to common recruitment, enrolment, and interview challenges [39]. Women were able to draw on a diverse array of lived experience to advise the team on where and how to advertise the study to reach women who are further marginalized from the research process and how to manage challenging interviews. This included, for instance, PRA-led training on how to recognize signs of drug withdrawal and how to support and engage a participant who is exhibiting signs of drug withdrawal.

*PRAs becoming study stewards in their communities and supporting the engagement of underserved women*. Through this process, PRAs became advocates for the study in their communities. In several settings, this is critical, as many women with HIV report being disenfranchised by the research process and are disinclined to participate. With PRAs engaged at every stage creating a study that is *by*, *with*, and *for* women with HIV, PRAs lend a trusted and insider voice to assure participants that “this study is different.” PRA involvement in CBR has been shown to increase engagement of typically underserved and harder-to-reach populations [29, 30].

*Stronger community connections* with ASOs, community-based organizations, policy-makers, other researchers and clinicians to help facilitate knowledge translation, advocacy, and action on formative study findings and processes. Such connections enhance opportunities for research findings to be effectively translated back into the community that it aims to serve.

### Recommendations

Drawing on the challenges and lessons learned through the process of hiring, training, and supporting a diverse team of women with HIV to become PRAs in CHIWOS, the following recommendations arose for teams hoping to develop similar teams in the future:

1. Commit seriously and rigorously to the training development and implementation processes, and the associated resource implications. This includes allotting sufficient time and resources for engaging women living with HIV in developing the training curriculum. This recommendation is particularly critical for training that aims to be inclusive of the diversity of linguistic, lived, and research experience among PRAs.
2. Create employment and training processes that are flexible and responsive to women’s needs and experiences, are reflective of hiring priorities and principles, and tailored to match regional contexts.
3. Allot sufficient resources for translation and cultural adaptation of training tools.
4. View PRA hiring, training, and support as iterative processes that continue throughout the life of the study, rather than as a one-off event that happens before data collection.
5. Integrate PRAs as training facilitators based on their skills, experiences, and expertise.
6. Financially compensate PRAs for completing the training and for any work performed as part of the study.
7. Include training outcomes that foster team building, trust, self-care, and communication.
8. View training as an opportunity to build both researcher and PRA capacity.

### Limitations

This paper has limitations. First, we were unable to determine the potential benefits and additional impacts of meaningful involvement for women with HIV beyond their capacity as a PRA. Second, despite our attempts to purposefully engage women who are typically underserved and under-researched, there is a constant need for reflection on how the community is defined. For example, women who were recruited as PRAs were already at least somewhat connected to a peer network, accessing services at organizations, and/or engaged in research, potentially excluding the most marginalized groups of women affected by HIV. Such engagement in community-based research practice demands constant reflexivity, and an understanding that there may be important perspectives sidelined from discussions through structural processes that must be continually considered and addressed.

## Conclusions

Studies that involve members of the target community are key to increasing the relevance and potential impact of research. In our experience engaging women living with HIV within a large, national quantitative cohort study, for women to participate and benefit from research, studies must foster inclusive, flexible, safe, and reciprocal approaches to peer employment, training, and support that is tailored to regional contexts and women’s lives. While our goal was to build research capacity among Peer Research Associates, our use of a collaborative strength-based training approach supported capacity building among all team members. We hope that our approach alongside our challenges, lessons learned, and recommendations can be both encouraging and useful to future studies committed to meaningfully engaging members of underserved communities in research.

## Data Availability

All PRA training materials are available in Open Access format (in both English and French) on our website: http://www.chiwos.ca/pra-training-materials/?doing_wp_cron=1548102397.6580440998077392578125&lang=en.

## Abbreviations

ART: Antiretroviral therapy
ASO: AIDS Service Organization
CBR: Community-based research
CHIWOS: Canadian HIV Women’s Sexual and Reproductive Health Cohort Study
GIPA: Greater Involvement of People living with HIV/AIDS
LGBTQ2S: Lesbian, gay, bi, trans, queer, or two spirit
MIPA: Meaningful Involvement of People living with HIV/AIDS
MIWA: Meaningful involvement of Women living with HIV/AIDS
PRA: Peer Research Associate

## Footnotes

1 In Quebec, all women who applied for a PRA position were hired.
